# Foot Arch Height and Quality of Life in Adults: A Strobe Observational Study

**DOI:** 10.3390/ijerph15071555

**Published:** 2018-07-23

**Authors:** Daniel López-López, Juan Manuel Vilar-Fernández, Gonzalo Barros-García, Marta Elena Losa-Iglesias, Patricia Palomo-López, Ricardo Becerro-de-Bengoa-Vallejo, Cesar Calvo-Lobo

**Affiliations:** 1 Research, Health and Podiatry Unit, Department of Health Sciences, Faculty of Nursing and Podiatry, Universidade da Coruña, 15403 Ferrol, Spain; daniellopez@udc.es (D.L.-L.); gonzalo_barros@yahoo.es (G.B.-G.); 2 Modeling, Optimization and Statistical Inference Research Group, Universidade da Coruña, 15071 A Coruña, Spain; juan.vilar@udc.es; 3 Faculty of Health Sciences, Universidad Rey Juan Carlos, 28922 Alcorcón, Spain; marta.losa@urjc.es; 4 University Center of Plasencia, Universidad de Extremadura, 10600 Plasencia, Spain; 5 Facultad de Enfermería, Fisioterapia y Podología, Universidad Complutense de Madrid, 28040 Madrid, Spain; ribebeva@ucm.es; 6 Nursing and Physical Therapy Department, Faculty of Health Sciences, Universidad de León, Ponferrada, 24401 León, Spain; ccall@unileon.es

**Keywords:** adult, flatfoot, foot disease, flat foot, quality of life

## Abstract

Background: Variations in the foot structure related with the high or low arch are identified common lower limb conditions, and it is supposed to be the effect on the quality of life (QoL) associated to foot health in adults. Here we aimed to determine the relationships between relatively high and low feet arches and QoL. Methods: A cross-sectional study was carried out. Among 138 adults enrolled in the study, 66 had a high arch, 21 had a low arch, and 51 were within the normal range. Changes related to the foot structure were analyzed using Area Calc version 2.6 software, and data obtained using the Foot-Health-Status-Questionnaire (FHSQ), whose domains were compared between foot arch groups by means of the one-way analysis of variance (ANOVA). Results: The results of the FHSQ comparison between the three groups within the sample population did not show any statistically significant difference (*p* > 0.05) for any domains of specific foot (pain, function, general health and footwear) and general (general health, physical activity, social function and vigor) health-related QoL. Conclusions: Specific foot and general health-related QoL did not seem to be influenced by the foot arch height between high, normal and low feet arches heights. Nevertheless, further studies with higher sample sizes and matched-paired groups should be carried out.

## 1. Introduction

The foot structure performs a key role in maintaining gait posture and balance. Therefore, most common variations in foot structure result in orthopedic conditions. Relatively high (cavus foot, talipes foot or pes cavus) or low (flatfoot or pes planus) medial longitudinal arches (MLAs) can result in musculoskeletal disorders affecting the lower limb and foot function [1,2,3,4].

Although the prevalence of foot structure problems has been reported as 46% to 80% in clinical practice and institutional settings [5,6], how foot structure problems affect functional impairment and foot and ankle pain in the adult population has been poorly studied. 

Furthermore, the MLA is a complex structure, whose development is influenced by sex, type of footwear, use of shoes, walking barefoot, and body mass index (BMI), which might affect balance, flexibility, and stability in the foot [7,8]. A decreased arch increase the risk of hallux valgus, hammer or overlapping toes, medial tibial stress syndrome, patellofemoral pain, metatarsalgia, hallux rigidus, Tailor’s bunion, and lower back pain [9,10,11,12]. An increased arch might lead to difficulty in putting on footwear, hammertoes, metatarsalgia, sesamoiditis, plantar heel pain, corns and keratosis, ankle sprains, and stress fractures [13,14,15,16].

These changes in foot structure are associated with high cost, increased economic burden, and represent a serious public health concern because of the necessity of surgery and associated pain in other regions (lower limb, hip, and knee), as well as gait disturbance, risk of falling, and neurological alterations [17,18,19].

The relationship between QoL and foot health problems in adults has been poorly studied. Here we aimed to determine whether relatively high and low feet arches affect quality of life (QoL). We hypothesized that variations in foot structure would negatively affect QoL.

## 2. Materials and Methods

### 2.1. Design and Sample

A cross-sectional study was carried out. A total of 138 adults (18–64-year-old) part in this descriptive observational investigation carried out at a private podiatric medicine & surgery center where give a treat of foot and ankle disorders in the city of A Coruña, localized in the northwest of Spain during thirteen months, since February 2016–March 2017.

Study participants were selected using a non-randomized and successive sampling technique. The mean age of the participants was 46.20 ± 11.37 years. The inclusion criteria were: >18-year-old; healthy adults without medical problems; and providing informed consent. The exclusion criteria were: Trauma in the lower limb, previous history of foot and limb surgery, neurological problems, autoimmune disease, loss of partial autonomy in daily life activities, pharmacotherapy, refusal to provide written consent, and inability to following the research guidelines.

### 2.2. Procedure

All podiatric medical examinations, measurements, and controls from recorded were made by an only the identical experienced medical podiatry using the same protocol. First, every subject was consulted about their medical status, including age, sex, actual and previous activities sporting, medical history and family history, and other conditions problems.

The second step was an anthropometric assessment of features, which included height, weight, and BMI [20].

Next, we determined the footprint of each participant using the photopodogram method [21]. This tool consists of painting the sole of the foot with paper developer liquid and asking each participant to stand in a bipedal position on white paper on a flat horizontal area for 60 s [22]. The footprints thus obtained were analyzed using AreaCalc v2.6 software [21]. This method has been shown to be reliable, with an intraclass correlation coefficient of 0.96–0.99 [21,23]. Next, following the protocol by Cavanagh and Rodgers, we determine the index arch in three types of the foot, which included low arch (<0.21), normal arch (0.21–0.26), and high arch (>0.26) [22]. In a final step, subjects were asked to complete the Foot-Health-Status-Questionnaire (FHSQ) [22]. This validated tool on QoL is intended to measure foot condition on a scale from zero (poorest) to one-hundred (best) [24]. Also, the FHSQ contains three sections, with four domains or subscales for each section. The first includes foot pain, foot function, footwear, and overall foot health. The second assesses general health, physical activity, social capacity, and vigor. The third collects socio-demographic data and medical records. This instrument recorded an upper degree validity with alfa of Cronbach 0.89–0.95 and upper retest reliability with an intraclass correlation coefficient of 0.74–0.92 [25]. 

### 2.3. Ethical Considerations

The Institutional Research and Ethical Committee at the public University of Coruña approved this study (C.E. 27/2016). We adhered to the principles of the Declaration of Helsinki always and all participants provided informed consent before being enrolled in our research.

### 2.4. Sample Size Calculation

The sample frame was analyzed using Clinical Epidemiology Research software, University of Coruña (http://www.fisterra.com/mbe/investiga/9muestras/9muestras2.asp) [26]. The statistical treatment was based on adults living in the city of Coruña, Northwest Spain, with an estimated population of ~150,000 adults.

Considering a two-tailed test, an alfa level of 0.05, a desired might analysis of 95% with a beta level of 20%, a precision of 4% for proportion of 50% (*p* = 0.5), and calculating a loss of healthy adults of 15%, we estimated that at least 114 subjects should be analyzed. A total of 138 adults people participated in this observational research.

### 2.5. Statistical Analysis

The IBM software package IBM SPSS statistical software (Version 22.0, IBM *Corp.*, Armonk, NY, USA) for Windows was used to analyze the data. Categorical data were shown as frequencies and percentages, while quantitative data were described as mean and standard deviation (SD), according to the Shapiro-Wilk normality test. For the quantitative variables, between-group comparisons were performed by the one-way analysis of variance (ANOVA) for independent samples. The Chi-Squared test (χ^2^) was used for categorical data. FHSQ scores were obtained from the FHSQ Data Analysis Software (Version 1.03, Care Quest, Brisbane, Australia). In all the analyses, *p* < 0.05 (with a 95% confidence interval) was considered statistically significant.

## 3. Results

A total of 138 participants completed all research stages. Demographic and social characteristics are shown in Table 1 and Table 2, respectively. No significant sociodemographic differences (*p* > 0.05) were found between groups, except for sex distribution (*p* < 0.001).

The results of the FHSQ comparison between the three groups within the sample population did not show any statistically significant difference *(p* > 0.05) and are shown in Table 3.

## 4. Discussion

Here we aimed to determine the relationship between having relatively high or low arched feet and QoL. This was achieved using a self-reported FHSQ in the adult population. Our alternative hypothesis has shown to be refused and the foot arch height (high, normal and low arch) did not seem to be related to QoL impairments related to the foot and overall health.

Previously to comparing the findings in this sections, it is worth showing that the various groups are meaningful in the adults and representative with anterior reports in the literature review that have associated with MLA with non-symptomatic foot [27], plantar pressure, and foot arch height [28,29], children and impact of MLA [30], concluding that changes in MLA can lead to health problems. 

By definition, the volunteers with various degrees of MLA were classified according to the protocol designed by Cavanagh and Rodgers [22] related to index arch, in agreement with previous studies that have analyzed these factors to show standard assessment of this condition and contributed to medical diagnostic and medical treatment of the foot problems [31,32].

Thus, the relationship between FHSQ values showed similar findings with the high, normal or low arch of the participants. Despite there were not statistically significant differences between foot arch height, in the first section present lower scores in the footwear and foot health domains suggesting that adults could experience more foot problems in terms of can putting shoes and believe that their feet are in a poor state of health, without differences as regards MLA [33,34,35,36], highlighting the importance of regular foot checks in this population.

Too, in the second section, this subjects presented lower points in the vigor may be related to restriction in the day a day to realizing sports activities and these findings are similar to other research.

This study has several limitations. Notably, this research resulted many people from another place in the world. A random sampling design would have increased the power of this study. Also, the impact of other cultural factors and the economic status on MLA-related QoL could have been included. Lastly, although a sample size calculation was carried out, consecutive sampling bias should be considered, and a simple randomization sampling process would be better for future studies. Finally, due to our sample showed statistically differences in sex distribution and the number of participants in each group was not balanced, further studies with higher sample sizes and matched-paired groups should be carried out. Therefore, variations in foot structure related to MLA may be an important issue and requires further research to identify the best interventions for preventing and controlling limb problems in this population.

## 5. Conclusions

Specific foot and general health-related QoL did not seem to be influenced by the foot arch height between high, normal and low feet arches heights. Nevertheless, further studies with higher sample sizes and matched-paired groups should be carried out.

## Figures and Tables

**Table 1:** Demographic characteristics of the sample population.

Demographic Characteristics	Total Group Mean (SD) 95% CI *N* = 138	Low Arch Mean (SD) 95% CI *N* = 66	Normal Arch Mean (SD) 95% CI *N* = 51	High Arch Mean (SD) 95% CI *N* = 21	One-Way Anova *p*-Value (F Statistic)
Sex, f/m (%)	106/32 (76.8/23.2) *	45/21 (62.8/31.8) *	48/3 (94.1/5.9) *	13/8 (61.9/38.1) *	<0.001 (13.955) **
Age (years)	46.20 (11.370) 44.29–48.12	47.29 (10.834) 44.25–50.34	44.85 (11.437) 42.04–47.66	47.81 (2.719) 42.14–53.48	0.726 (0.864)
Weight (kg)	71.359 (14.478) 68.922–73.796	67.696 (13.600) 66.983–65.000	72.273 (14.724) 68.653–75.892	77.381 (13.912) 71.047–83.715	0.080 (1.453)
Height (cm)	1.661 (0.092) 1.645–1.676	1.644 (0.088) 1.620–1.669	1.670 (0.093) 1.645–1.692	1.674 (0.097) 1.630–1.719	0.111 (1.384)
BMI (kg/m^2^)	25.756 (4.148) 25.058–26.454	24.940 (4.041) 23.803–26.076	25.817 (4.187) 24.788–26.847	27.546 (3.871) 25.784–29.308	0.604 (1.004)

Abbreviations: ANOVA, analysis of variance; BMI, body mass index; f, female; SD, standard deviation. In all the analyses, *p* < 0.05 (with a 95% confidence interval) was considered statistically significant. * Frequencies and percentages were used. ** Chi-squared test and χ^2^ statistic were used.

**Table 2:** Social characteristics of the sample population.

SocioDemographic Characteristics	Subcategory	Total Group *n* (%) *N* = 138	Low Arch *n* (%) *N* = 51	Normal Arch *n* (%) *N* = 66	High Arch *n* (%) *N* = 21	Chi-Squared *p*-Value (χ^2^)
Education level	I. primary	5 (3.6)	1 (0.7)	2 (1.4)	2 (1.4)	0.890 (3.613)
	C. primary	25 (18.1)	9 (6.5)	12 (8.7)	4 (2.9)	
	Secondary	42 (30.4)	16 (11.6)	20 (14.5)	6 (4.3)	
	Degree	43 (31.2)	18 (13.0)	20 (14.5)	5 (3.6)	
	S. degree	23 (16.7)	7 (5.1)	12 (8.7)	4 (2.9)	
Professional activity	Student	2 (1.4)	2 (1.4)	0 (0.0)	0 (0.0)	0.112 (12.996)
	freeland	25 (18.1)	6 (4.3)	16 (11.6)	3 (2.2)	
	employed	92 (66.7)	39 (28.3)	39 (28.3)	14 (10.1)	
	unemployed	10 (7.2)	4 (2.9)	4 (2.9)	2 (1.4)	
	Retired	9 (6.5)	0 (0.0)	7 (5.1)	2 (1.4)	
Civil status	Single	19 (13.8)	7 (5.1)	8 (5.8)	4 (2.9)	0.413(8.207)
	divorced	9 (6.5)	3 (2.2)	5 (3.6)	1 (0.7)	
	widowed	6 (4.3)	4 (2.9)	2 (1.4)	0 (0.0)	
	couple	35 (25.4)	16 (11.6)	17 (12.3)	2 (1.4)	
	married	69 (50.0)	21 (15.2)	34 (24.6)	14(10.1)	

Abbreviations: C, complete; I, incomplete; S, superior. In all the analyses, *p* < 0.05 (with a 95% confidence interval) was considered statistically significant.

**Table 3:** Comparisons of F.H.S.Q scores for the cases and control groups.

Domains F.H.S.Q	Total Group Mean (SD)9 5% CI *N* = 138	Low Arch Mean (SD) 95% CI *N* = 66	Normal Arch Mean (SD) 95% CI *N* = 51	High Arch Mean (SD) 95% CI *N* = 21	One-Way Anova *p*-Value (*F* Statistic)
Foot pain	70.99 (23.18) 67.09–74.89	67.44 (25.41) 60.29–74.59	73.16 (21.52) 67.87–78.45	72.77 (22.58) 62.49–83.05	1.481 0.070
Foot function	77.49 (27.79) 72.81–82.17	74.39 (29.61) 66.06–82.72	71.23 (26.50) 71.23–84.26	84.23 (27.29) 71.80–96.65	1.047 0.439
Footwear	46.62 (30.18) 41.54–51.70	41.67 (28.92) 33.53–49.80	46.46 (32.09) 38.57–54.35	59.13 (23.99) 48.20–70.05	0.899 0.673
Foot health	48.17 (27.00) 43.63–52.72	42.84 (27.59) 35.08–50.60	51.71 (26.89) 45.10–58.31	50.00 (24.98) 38.63–61.37	1.244 0.207
General health	65.65 (23.71) 61.66–69.64	65.88 (26.09) 58.54–73.22	66.67 (22.42) 61.16–72.18	61.90 (22.27) 51.77–72.04	0.997 0.515
Physical activity	82.45 (23.21) 78.54–86.35	77.12 (28.28) 69.17–85.08	85.27 (20.10) 80.33–90.21	86.51 (16.16) 79.15–93.86	1.297 0.164
Social function	81.43 (21.79) 77.76–85.10	79.17 (24.96) 72.15–86.19	82.58 (20.32) 77.58–87.57	83.33 (18.26) 75.02–91.64	0.857 0.738
Vigor	52.26 (20.35) 48.84–55.69	50.74 (22.00) 44.55–56.92	53.31 (17.55) 49.00–57.63	52.68 (24.81) 41.39–63.97	1.381 0.112

Abbreviations: F.H.S.Q., Foot Health Status Questionnaire; SD, standard deviation. In all the analyses, *p* < 0.05 (with a 95% confidence interval) was considered statistically significant.
